# Complete Genome Sequence of a Porcine Epidemic Diarrhea Virus from a Novel Outbreak in Belgium, January 2015

**DOI:** 10.1128/genomeA.00506-15

**Published:** 2015-05-21

**Authors:** Sebastiaan Theuns, Nádia Conceição-Neto, Isaura Christiaens, Mark Zeller, Lowiese M. B. Desmarets, Inge D. M. Roukaerts, Delphine D. Acar, Elisabeth Heylen, Jelle Matthijnssens, Hans J. Nauwynck

**Affiliations:** a Ghent University, Faculty of Veterinary Medicine, Department of Virology, Parasitology and Immunology, Laboratory of Virology, Merelbeke, Belgium; b KU Leuven–University of Leuven, Department of Microbiology and Immunology, Laboratory for Clinical and Epidemiological Virology, Rega Institute for Medical Research, Leuven, Belgium

## Abstract

Porcine epidemic diarrhea virus (PEDV) is a member of the family *Coronaviridae* and can cause severe outbreaks of diarrhea in piglets from different age groups. Here, we report the complete genome sequence (28,028 nt) of a PEDV strain isolated during a novel outbreak in Belgium.

## GENOME ANNOUNCEMENT

Porcine epidemic diarrheic virus (PEDV) caused outbreaks of diarrhea in Europe from the 1970s to the 1990s, but has since been reported only sporadically in the last few decades (1, 2). From 2010, severe outbreaks with mortality have been identified in Asia, and since the spring of 2013, the virus was detected for the first time in U.S. swine herds (3–9). Genetically, these American isolates were highly related (99.5% nucleotide similarity) to Asian PEDV strains (8, 10). In January 2014, a novel PEDV variant strain OH851 was isolated in the United States, which was associated with milder disease symptoms (11). Similar strains contained several deletions and insertions in their spike genes and were therefore called INDEL strains (6). In May 2014, an outbreak of diarrhea occurred in fattening pigs on a German farm, and the isolated strains were genetically almost identical to U.S. strain OH851 (12).

An outbreak of diarrhea in fattening pigs occurred on a Belgian pig farm at the end of January 2015, and the presence of PEDV RNA in the feces of these pigs was demonstrated using an in house RT-qPCR assay targeting the nucleocapsid gene. This was the first confirmed PEDV case in Belgium in decades. Therefore, the complete genome of this novel isolate, BEL/15V010/2015, was unraveled by next-generation sequencing, in order to assess its genetic relation to other PEDV isolates circulating around the globe. Viral particles were purified, RNA was extracted using the QIAamp Viral RNA mini kit (Qiagen), and whole transcriptome amplification was performed (WTA2 kit, Sigma Aldrich) to generate cDNA. The sequencing library was prepared using the Nextera XT DNA library preparation kit (Illumina). Sequencing was performed on a HiSeq 2500 platform (Illumina) for 301 cycles (150-bp paired-end reads). Raw reads were trimmed for quality and adapters using Trimmomatic (13), and reads were mapped using BWA against the German PEDV strain L00721 (GenBank LM645057) (14). Remaining gaps were closed using Sanger sequencing.

Isolate 15V010 contained a genome with a size of 28,028 nucleotides (nt), excluding the poly-A tail. Open reading frame 1 (ORF1), encoding the replicase polyprotein, was located between nt 293 and 12601 and between nt 12601 and 20637, with a ribosomal frameshift between both parts. The other genes were arranged as follows: spike (S), nt 20634 to 24785; accessory membrane protein (mp, ORF3), nt 24785 to 25459; envelope (E), nt 25440 and 25670); membrane (M), 25678 to 26358; and nucleocapsid (N), nt 26370 to 27695.

At the whole-genome level, strain 15V010 was most closely related to strain L00721 (99.9% nucleotide similarity) isolated in Germany in 2014, and the prototype U.S. INDEL strain OH851 (99.4%). Strain 15V010 was less closely related to the former prototype European strain CV777 (97.0%).

In conclusion, a PEDV strain from a novel outbreak in Belgium was characterized and showed high relatedness to recent German PEDV isolates and U.S. PEDV INDEL strains. Our findings indicate that PEDV is still or again circulating in Europe. Surveillance and development of prophylactic measures are urgently needed.

### Nucleotide sequence accession number. 

The complete genome sequence of PEDV BEL/15V010/2015 has been deposited at GenBank under the accession number KR003452.
